# Methylene blue as adjunctive therapy in septic shock: correct drug diluent derives optimal efficacy

**DOI:** 10.1186/s13054-023-04615-2

**Published:** 2023-08-29

**Authors:** Dilip Dubey, Pawan Ray, Ali Imran

**Affiliations:** Institute of Critical Care Medicine, Medanta Hospital, Lucknow, Uttar Pradesh India

We read with a great interest, the randomized controlled trial “Early adjunctive methylene blue in patients with septic shock” published by Ibarra-Estrada et al. in Critical care recently [1]. We would like to congratulate all the authors of the study for addressing such an important aspect in critical care and trying to answer the research question by conducting a randomized controlled trial.

The authors have concluded that the use of methylene blue (MB) within 24 h in patients with septic shock reduces the duration of vasopressor use, cumulative fluid balance, hospital and intensive care unit stay without significant adverse events as compared to placebo.

In methodology, authors mentioned that patients assigned to MB group received an intravenous (IV) infusion of 100 mg MB in 500 ml of 0.9% sodium chloride solution [1].

We would like to highlight that since MB is a hypotonic solution with osmolarity of about 10–15 mOsm/kg, dilution with 0.9% sodium chloride solution, could lead to precipitation of MB, as the presence of chloride (Cl^−^) ions reduces the solubility of MB [2–4]. Precipitation of a drug in a solution can reduce its therapeutic efficacy due to drug inactivation, can occlude the infusion lines, may cause thrombophlebitis or rarely particulate embolization and multiple organ dysfunction [5].

Therefore, to avoid precipitation of MB in a solution and to improve its clinical efficacy without adversely affecting the patient, we suggest that MB should be diluted in either a sterile water or 5% dextrose solution before intravenous administration.

## Data Availability

Not applicable.
